# Genome-wide association study reveals *BET1L* associated with survival time in the 137,693 Japanese individuals

**DOI:** 10.1038/s42003-023-04491-0

**Published:** 2023-02-03

**Authors:** Masato Akiyama, Saori Sakaue, Atsushi Takahashi, Kazuyoshi Ishigaki, Makoto Hirata, Koichi Matsuda, Yukihide Momozawa, Yukinori Okada, Toshiharu Ninomiya, The Biobank Japan project, Masaru Koido, Takayuki Morisaki, Akiko Nagai, Yoji Sagiya, Chikashi Terao, Yoshinori Murakami, Michiaki Kubo, Yoichiro Kamatani

**Affiliations:** 1 Laboratory for Statistical Analysis, RIKEN Center for Integrative Medical Sciences, Yokohama, 230-0045 Japan; 2 Laboratory for Statistical and Translational Genetics, RIKEN Center for Integrative Medical Sciences, Yokohama, 230-0045 Japan; 3 Department of Ocular Pathology and Imaging Science, Graduate School of Medical Sciences, Kyushu University, Fukuoka, 812-8582 Japan; 4 Department of Statistical Genetics, Osaka University Graduate School of Medicine, Suita, 565-0871 Japan; 5 Department of Genomic Medicine, Research Institute, National Cerebral and Cardiovascular Center, Osaka, 564-8565 Japan; 6 Laboratory of Genome Technology, Human Genome Center, Institute of Medical Science, The University of Tokyo, Tokyo, 108-8639 Japan; 7 Laboratory of Clinical Genome Sequencing, Department of Computational Biology and Medical Sciences, Graduate school of Frontier Sciences, The University of Tokyo, Tokyo, 108-8639 Japan; 8 Laboratory for Genotyping Development, RIKEN Center for Integrative Medical Sciences, Yokohama, 230-0045 Japan; 9 Laboratory for Systems Genetics, RIKEN Center for Integrative Medical Sciences, Yokohama, 230-0045 Japan; 10 Department of Epidemiology and Public Health, Graduate School of Medical Sciences, Fukuoka, 812-8582 Japan; 11 Division of Molecular Pathology, The Institute of Medical Science, The University of Tokyo, Tokyo, 108-8639 Japan; 12 RIKEN Center for Integrative Medical Sciences, Yokohama, 230-0045 Japan; 13 Laboratory of Complex Trait Genomics, Department of Computational Biology and Medical Sciences, Graduate School of Frontier Sciences, The University of Tokyo, Tokyo, 108-8639 Japan; 14 Department of Public Policy, Institute of Medical Sciences, The University of Tokyo, Tokyo, Japan; 15 Laboratory of Genome technology, Institute of Medical Sciences, The University of Tokyo, Tokyo, Japan

**Keywords:** Genome-wide association studies, Prognostic markers

## Abstract

Human lifespan is reported to be heritable. Although previous genome-wide association studies (GWASs) have identified several loci, a limited number of studies have assessed the genetic associations with the real survival information on the participants. We conducted a GWAS to identify loci associated with survival time in the Japanese individuals participated in the BioBank Japan Project by carrying out sex-stratified GWASs involving 78,029 males and 59,664 females. Of them, 31,324 (22.7%) died during the mean follow-up period of 7.44 years. We found a novel locus associated with survival (*BET1L*; *P* = 5.89 × 10^−9^). By integrating with eQTL data, we detected a significant overlap with eQTL of *BET1L* in skeletal muscle. A gene-set enrichment analysis showed that genes related to the *BCAR1* protein–protein interaction subnetwork influence survival time (*P* = 1.54 × 10^−7^). These findings offer the candidate genes and biological mechanisms associated with human lifespan.

## Abstract (web summary)

A genome-wide association study in the BioBank Japan cohort reveals new locus, *BET1L*, which is associated with survival time in a Japanese population.

## Introduction

Human lifespan, is reported to be a heritable trait^1^. Genome-wide association studies (GWASs) have suggested several genetic loci associated with lifespan^2–10^. However, few of these studies assessed the associations of genetic variants with survival information. Due to the difficulty to follow up the participants to collect a sufficient numbers of death information, an approach using parental lifespan as phenotype and the genotypes of their children as explanatory variables was used to investigate the genetic architecture of human lifespan. Moreover, the majority of GWASs for lifespan involved European populations; investigations in Asian populations are limited to a Chinese study with a small sample size^11^. Therefore, a large-scale GWAS of a non-European population with a long follow-up is needed to identify novel loci associated with lifespan.

We analyzed Japanese individuals who participated in the BioBank Japan (BBJ) project^12^, which contains follow-up data of >140,000 individuals; >35,000 individuals died during the >8-year follow-up survey^13^. Furthermore, information on 47 disease status—including cardiovascular, metabolic, autoimmune, and malignant diseases—is available^14^. By utilizing these large-scale follow-up data from an Asian population, we conducted a GWAS to identify genetic loci associated with lifespan in Japanese individuals and to provide biological insight into human lifespan. We identified a locus showing a genome-wide significant association. Finally, a gene-set enrichment analysis identified genes related to survival duration.

## Results

### Genome-wide association study

To identify loci associated with lifespan in the Japanese population, we performed a GWAS for survival time after their registration in the BBJ project using a total of 137,693 individuals (78,029 males and 59,664 females). The baseline characteristics of the participants are summarized in Supplementary Tables 1, 2. Among the participants, 31,324 individuals (22.7%) died (causes of death are summarized in Supplementary Table 3) during the mean follow-up of 7.44 years. By considering survival prognoses and that males and females are affected by sex-specific diseases, such as gynecological cancers and prostate cancer, we performed sex-stratified GWASs using 6,108,833 single nucleotide polymorphisms (SNPs), which were imputed into the East Asian (EAS) samples of the 1000 Genomes Project (1KG)^15^.

**Fig. 1:**
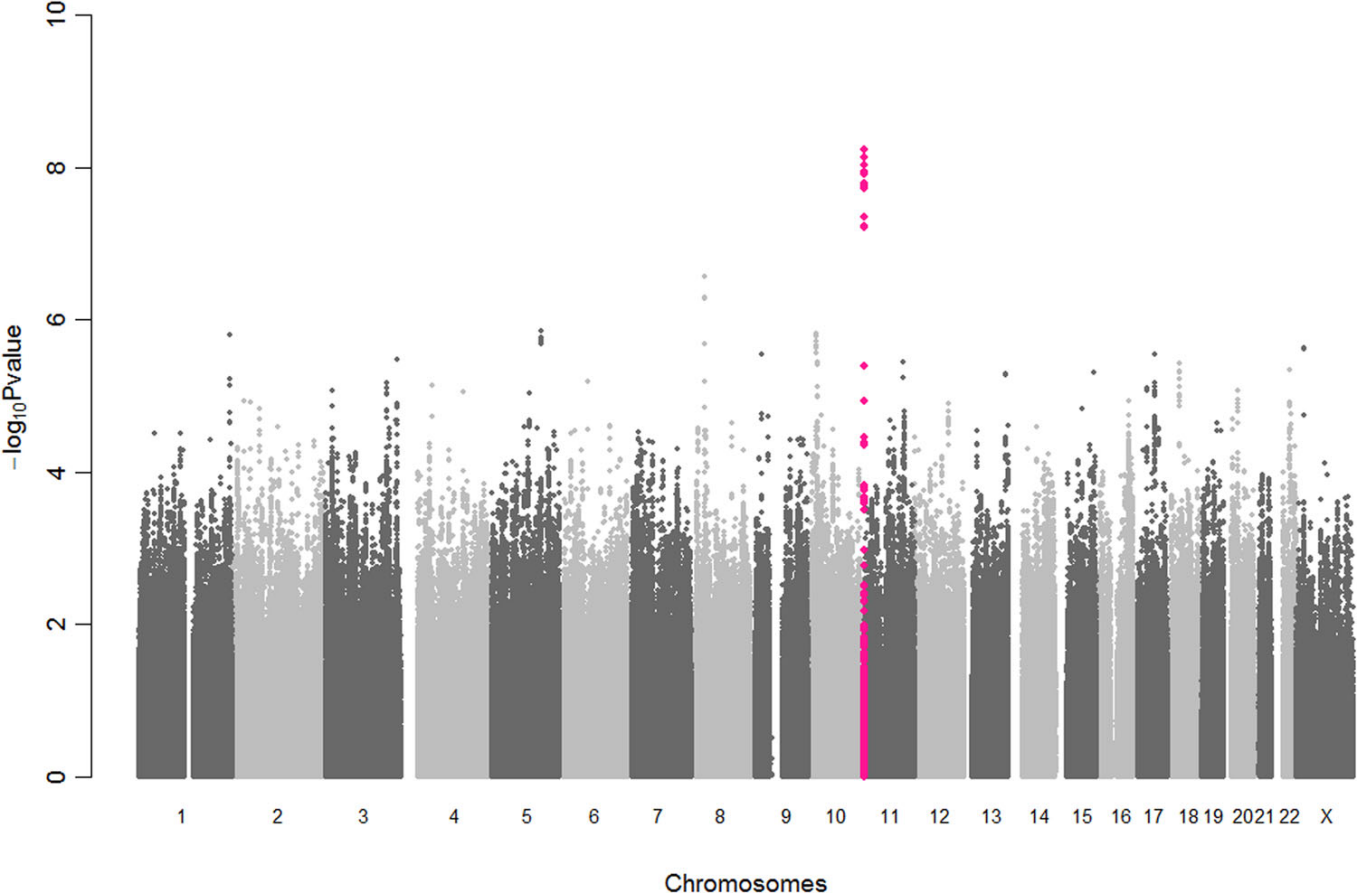
Manhattan plot of the meta-analysis of sex-stratified GWAS. Associations of the meta-analysis of sex-stratified GWASs. *Y*-axis, *P*-value on a−log10 scale. *X*-axis, chromosomal position based on NCBI build 37. Variants near the newly identified *BET1L* locus are highlighted in pink.

**Fig. 2:**
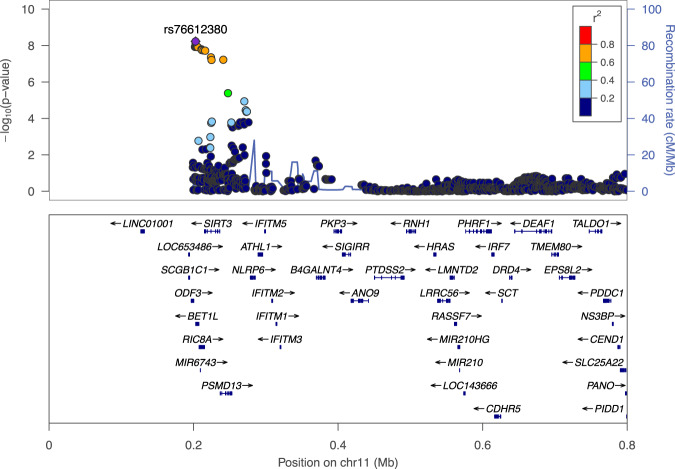
Regional association plot of *BET1L* region. Associations of the *BET1L* region by LocusZoom software (v. 1.3). Colored according to the linkage disequilibrium with the lead variant (rs76612380).

The genomic inflation factors (lambda GC) were 1.097 and 1.065 for males and females, respectively (Supplementary Fig. 1). Nevertheless, when we evaluated the associations using SPACox software^16^, the genomic inflation factors were decreased to the acceptable range (lambda GC = 1.029 and 1.004 for male and female, respectively), suggesting inflated statistics were not due to population stratification or cryptic relatedness. Therefore, we did not apply GC correction to each dataset. In the sex-stratified GWASs, no locus satisfied genome-wide significance in either dataset (Supplementary Fig. 2). After combining the results, we observed a significantly associated locus with genome-wide significance (Figs. 1, 2; rs76612380; *P*_meta_ = 5.89 × 10^−9^, hazard ratio [HR] = 0.92, 95% confidence interval [CI] = 0.89–0.95). The effect sizes of rs76612380 were similar between the sexes (Table 1; *P* for heterogeneity = 0.77).

**Table 1:** Meta-analysis of the sex-stratified GWASs.

SNP	CHR: POS	Study	No. of samples	RAF	HR	95% CI	*P*-value	
	Allele [REF/ALT]						SPACox	Wald test
rs76612380	11: 202,785	Male	78,029	0.90	0.92	0.89–0.95	2.15 × 10^−6^	7.99 × 10^−7^
	[T/C]	Female	59,664	0.90	0.93	0.88–0.97	2.84 × 10^−3^	1.95 × 10^−3^
		Combined	137,693	0.90	0.92	0.89–0.95		5.89 × 10^−9^

Position is based on NCBI build 37. Associations calculated using the Cox proportional hazard model with the ‘Survival’ package in R. Meta-analysis performed by the inverse variance method in each sex-stratified GWAS.

*CHR* chromosome, *POS* position, *RAF* reference allele frequency, *HR* hazard ratio (effect size of alternative allele), *CI* confidence interval.

**Fig. 3:**
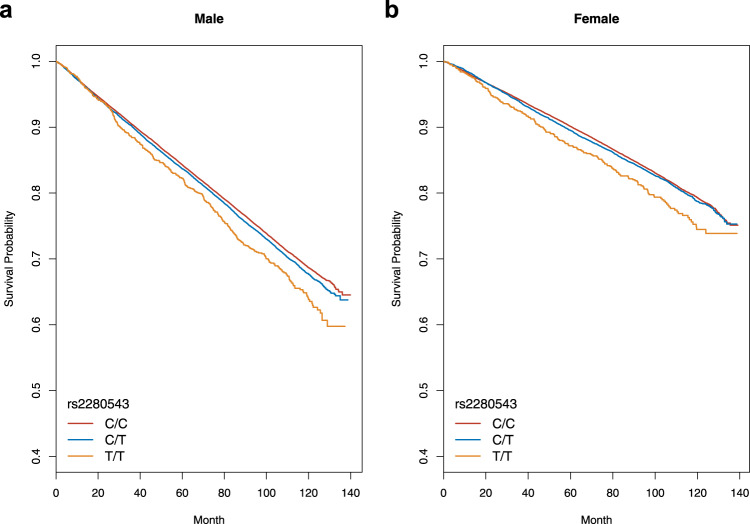
Survival curves stratified by rs2280543 genotypes in each sex. Survival curves plotted according to rs2280543 genotype in males (**a**) and females (**b**).

A survival curve generated using the genotyped proxy SNP (rs2280543; *P*_meta_ = 1.17 × 10^−8^; Pearson’s *r*^2^ between rs2280543 genotype and rs76612380 imputed dosage = 0.98) is shown in Fig. 3. In both males and females, T-allele homozygous individuals had a poorer prognosis than those of other genotypes, suggesting that part of the genetic effect is due to non-additive effect. We evaluated the non-additive effect of rs2280543 and confirmed its statistical significance (*P* = 0.02 in the sex-combined dataset; Supplementary Fig. 3).

The publicly available summary statistics of a large-scale GWAS of lifespan in a European population^9^ denoted that the rs2280543 T allele was associated with shorter parental lifespan (*P* = 5.10 × 10^−3^, *β* = −0.03 years, standard error [SE] = 0.01), although an association of rs76612380 was not reported. Next, we tested the association between rs2280543 and age at death in 31,324 individuals who died in our dataset and found a significant association in the same direction as effect size (*P* = 1.75 × 10^−3^, *β* = −0.36 years, SE = 0.12; Supplementary Fig. 4). Summary statistics from a recently reported meta-GWAS for longevity^10^ also included rs2280543 but the association result was not statistically significant (*P* = 0.18 and 0.14 for surviving to the 90th/99th percentile age, respectively).

### Functional annotation and pleiotropy of *BET1L*

To assess the biological role of the identified locus, we annotated variants in linkage disequilibrium (LD) with rs76612380 using HaploReg (v. 4.1)^17^. The variant was common in EAS samples (minor allele frequency [MAF] = 0.11) but rare (MAF < 5%) in all non-East Asian populations of 1KG. We also looked up gnomAD^18^ and confirmed that rs76612380 is rare in all registered non-East Asian populations. rs76612380 overlapped with the promoter histone marks of blood cells, and with the enhancer histone marks across nine tissues (Supplementary Table 4).

According to the GWAS catalog^19^, rs2280543 is reported to be associated with uterine fibrosis and intracranial aneurysm^20,21^. We also assessed pleiotropic effects of this locus in the catalog of genetic associations in the BBJ project across 220 traits^22^ using PheWeb^23^. Since statistics of rs76612380 were not available for all traits, we looked up the associations of rs2280543 with 219 traits, and found that 17 traits were significantly associated after Bonferroni correction (α = 2.28 × 10^−4^ [=0.05/219]; Supplementary Fig. 5 and Supplementary Table 5). Briefly, the associated traits are red blood cell related traits (red blood cell counts, hemoglobin, hematocrit, mean capsular volume, and mean capsular hemoglobin), cerebrovascular and cardiovascular diseases (cerebral aneurysm, subarachnoid hemorrhage, angina pectoris, and myocardial infarction), and others (uterine fibroid, colorectal cancer, cataract, compression fracture, alkaline phosphatase, cataract, drugs for peptic ulcer and gastro-esophageal reflux disease, and vasodilators used in cardiac diseases). Of these traits, 14 traits (except for alkaline phosphatase, compression fracture and myocardial infarction) showed significant overlaps (*r*^2^ > 0.7 between rs2280543 and each lead variant; Supplementary Table 5).

Next, we looked up the eQTL in the GTEx database^24^ (v6); rs76612380 is an eQTL of *BET1L* in skeletal muscle (Supplementary Fig. 6). Also, the top hit of this eQTL (rs79902640) was in strong LD with rs76612380 (*r*^*2*^ = 0.79 and 0.89 in EAS and EUR of 1KG, respectively). Although we searched the eQTLs of five subsets of immune cells in the Japanese population^25^, we did not find any significant overlaps between association peaks (*r*^*2*^ < 0.7; false discovery rate <5%). We also searched for eQTL effects of variants in LD with rs76612380 across multiple databases using HaploReg; however, only eQTL for *BET1L* in muscle skeletal was reported. These results suggest that the identified locus influences survival times by modulating *BET1L* expression in skeletal muscle. We also investigated truncated variants of *BET1L* using the GTEx portal and found rs3782121 (*r*^2^ with rs79902640 = 0.98) was suggested to have a splice disruption effect^26^ in skeletal muscle (posterior probability = 0.191).

### Relevance of *BET1L* signal to disease status

Since the BioBank Japan was composed of the hospital-based samples, we analyzed individuals with diseases that could confer a risk of death at baseline, including cardiovascular diseases and cancers. To account for the possibility that disease status might be a confounding factor, we conducted a sensitivity analysis by stratifying participants of each sex based on disease status. The result suggested that the identified locus influenced survival irrespective of disease status at baseline (Supplementary Fig. 7 and Supplementary Data 1).

Next, we evaluated the hypothesis that identified loci confer a risk of a fatal disease. We divided individuals who died (*N* = 31,324) into five categorized causes of death and investigated the difference after stratifying by proxy SNP (rs2280543). There was no significant difference between causes of death and rs2280543 genotypes (Supplementary Fig. 8; *P* for chi-square test = 0.29). We also considered the pleiotropic effects of *BET1L*, and performed a sensitivity analysis after excluding the individuals who died due to cerebrovascular and cardiovascular diseases (*N* = 6088). We confirmed that the strong association of rs76612380 remained (Supplementary Fig. 9; *P* = 1.40 × 10^−7^, HR = 0.92, 95%CI: 0.89–0.95). Taken together, we interpreted that the effect of *BET1L* is not related to death from a specific disease category.

### Associations at previously reported loci

We next evaluated the associations of reported lifespan-associated loci (Supplementary Data 2); only SNPs that determines apolipoprotein e (*APOE*) alleles showed a significant association after Bonferroni correction (*P* = 2.50 × 10^−4^ and 5.95 × 10^−4^, HR = 0.95 and 1.07, 95% CI = 0.92–0.98 and 1.03–1.12, for rs429385 and rs7412, respectively) (α = 0.001 [=0.05/49]). Notably, we found significant sex-heterogeneity for rs429385 (HR = 0.97 and 0.90 for males and females, respectively; *P*_het_ = 0.02). We also evaluated the association of *APOE* haplotypes (Supplementary Fig. 10 and Supplementary Table 6). Compared to the previous study^27^, the effect size of ε2 was similar (HR = 0.94, 95%CI = 0.90–0.98); however, that of ε4 was weaker (HR = 1.05, 95%CI = 1.02–1.08; 95%CI were not overlapped with that of previous study [95% CI = 1.12–1.21]). We note that effect sizes of ε4 was different between sexes (*P*_het_ = 0.01). Therefore, genetic variants of *APOE* are associated with lifespan across populations.

### Gene-set enrichment analysis

To expand biological knowledge on human lifespan, we performed a pathway analysis using PASCAL^28^ with reconstituted gene sets implemented in DEPICT^29^. There was significant enrichment of genes in the *BCAR1* protein–protein interaction (PPI) subnetwork (*P* = 7.00 × 10^−8^, false discovery rate [FDR] = 1.01 × 10^−3^, Supplementary Data 3 and Supplementary Data 4). By considering that *BCAR1* encodes breast cancer anti-estrogen resistance 1, we hypothesized that the enrichment of the BCAR1 *PPI* subnetwork might differ in patients with cancers. We targeted five cancers with sample size more than 3,000, and performed gene-set enrichment analysis in the same way; however, we did not observe significant enrichment of BCAR1 PPI subnetwork (Supplementary Table 7), suggesting that significant enrichment of the gene-set was not driven by specific cancers.

## Discussion

In the present study, we performed a large-scale GWAS for survival time after the registration of the Biobank Japan project, and identified a locus associated with survival time in the Asian population at a genome-wide significant level. By utilizing the GWAS result, we revealed that genes included in *BCAR1* PPI subnetwork may influence human lifespan.

This study has two advantages in comparison with previous works. One is that we investigated the impact of genotypes on lifespan using participants’ information, including survival duration and disease status. The other is that we adjusted for participants’ disease status at baseline in the association study. Compared to prior works, which investigated the association of genotype using parental survival information as a surrogate^8,9^, our approach is more straightforward. Although a recent study suggested the usefulness of familial information for phenotype in genetic association studies, use of parental survival information is thought to reduce the effective sample size^9^. Furthermore, the disease status of participants was not considered in the association analyses in previous studies. This resulted in uncovering of genetic loci associated with life-threatening diseases such as cardiovascular, autoimmune, and smoking-related diseases and cancers as being associated with lifespan^9,10^. By contrast, *BET1L* was not associated with major causes of death in the Japanese population, suggesting that a non-disease-related biological mechanism underlies the locus.

Integrating the association signals of *BET1L* with eQTL data of the GTEx project, indicated a significant overlap between the GWAS signals and the eQTL of *BET1L* in skeletal muscle, suggesting that differences in *BET1L* expression influence survival duration. *BET1L* encodes Bet1 Golgi vesicular membrane trafficking protein-like protein. *BET1L* is reported to be a vesicle-associated SNARE^30^ and a pathway analysis in a recent large-scale GWAS for lifespan^9^ showed significant enrichment of genes belonging to vesicle-mediated transport. Therefore, our findings, together with those derived from European populations, implied that genes relevant to vesicular-mediated proteins affect human lifespan. Nevertheless, our functional annotation showed that the identified association signal was overlapped with chromatin marks of various tissues and cell-types. Therefore, we could not conclude that *BET1L* is a causative gene for the observed genetic associations. Further functional investigations are warranted to interpret the biological roles of this locus on human lifespan.

To our knowledge, our GWAS is the largest such study of human lifespan in an Asian population. Although the identified variant rs76612380 is common in East Asians, it is present in non-East Asian populations at low frequencies. Given that associations of the newly identified locus in the previous studies of European were weak, the allele frequency difference might influence this observation. Furthermore, considering this locus has a dominance effect, uncovering its associations is difficult because C-allele homozygotes are very rare in non-East Asian populations. This supports the advantage of investigating diverse populations to expand our knowledge on genetic architecture of lifespan. It should be also noted that most previously reported loci were not replicated in our GWAS. Given that some proportion of the previously reported loci have pleiotropic effects on diseases, possible explanation for this observation would be that we adjusted for disease status of the participants in the association analyses. On the other hand, we found a significant association at *APOE* locus. Considering together, our result suggests *APOE* could affect human lifespan regardless of disease status and ethnicities.

We observed a statistically significant non-additive effect of the identified locus. As shown in Fig. 3, the survival curve for the individuals with rs2280543 T-allele homozygote showed departure from that of the other genotypes (C/T and T/T) in both sexes. Although exact causes of non-additive effect have not been fully elucidated, we speculate that causal variant of the identified locus may have disruptive effect for a true causal gene as those in recessive disorders. Despite low posterior probability (0.191), rs3782121 was implied to have a splice disruption effect in skeletal muscle according to the GTEx project. Taken together, although functional evaluation is warranted, disruption of *BET1L* might induce non-additive effect.

Our results implied that the BCAR1 PPI subnetwork is associated with lifespan in the Japanese population. *BCAR1* encodes breast cancer anti-estrogen resistance protein 1. Because the applied gene sets were originally constructed based on predicted function and reconstituted gene sets^29^, the biological role of the identified gene set was unclear. Although further study is needed to link genes in the BCAR1 PPI subnetwork to survival duration, our results offer candidate genes and biological mechanisms relevant to human lifespan.

This study has several limitations. First, we did not include a replication set. Although the European GWAS supported our findings, further evaluation is needed. Second, the participants in the survival survey in the BBJ project had various systemic diseases. Therefore, evaluation of the impact of rs76612380 in the general population is warranted. Third, we observed non-negligible inflation in GWAS statistics when we used Wald test, whereas our previous analyses using the same dataset^31,32^ showed no such inflation as a result of population stratification and cryptic relatedness based on the LD score regression results. Given that observed inflations were decreased by using SPACox software, we considered that observed inflation might be due to Wald test as discussed in the recent study^27^. We note that the association of lead variant (rs76612380) satisfied genome-wide significance even if we applied single GC correction (*P*_GC_ = 2.39 × 10^−8^).

In conclusion, we performed a large-scale GWAS for survival time in the Japanese population and identified a novel locus, *BET1L*. Integrative analysis with eQTL data suggested that a change in *BET1L* expression in skeletal muscle influences survival time. These findings provide biological insight into the human lifespan.

## Methods

### Participants

We used the follow-up data of the BBJ project^12^. This follow-up survey from 2010 to 2014 included 141,612 participants with 32 systemic diseases at the time of participation^13^. We included participants genotyped by DNA genotyping array. We excluded individuals <20 years old and participants with amyotrophic lateral sclerosis. After standard quality control of genotype data (described below), we included 137,693 individuals in the analysis. We obtained written informed consent from all participants. This study was approved by the Ethics Committees of the participating hospitals, the University of Tokyo, and RIKEN.

### Genotyping and whole-genome imputation

We genotyped subjects using the Illumina HumanOmniExpressExome BeadChip or a combination of the Illumina HumanOmniExpress BeadChip and Illumina HumanExome BeadChip. We excluded individuals with a call rate <98%, closely related subjects estimated by identity-by-state analysis via visual inspection, and outliers in principal component analyses estimated using in-house software^31,32^.

We phased genotypes using MACH^33^ and imputed the variants using Minimac (v0.1.1). The phased genotype data of EAS samples from 1KG (phase1v3) were used as reference genotype information. We used the variants with a high imputation quality score (Rsq ≥ 0.7) in the association analysis. According to the heterozygosity of X-chromosomal variants, we excluded three males from the X-chromosomal analysis considering possible sex-errors.

### Association analysis

We performed GWAS using allelic dosage of imputed variants with a Cox proportional hazard model under the assumption of the additive genetic model using the ‘Survival’ package in R. We used age, age squared, sex, top 10 principal components, and affected diseases at baseline (Supplementary Table 1) as covariates. Two-sided *P*-values were used throughout the analyses. The dominance effect of the associated variants was evaluated by adding a dominance term to the additive genetic model. In order to evaluate possible statistical inflation resulted from Wald test, we evaluated associations of genetic variants estimated by saddle point estimation using SPACox software^17^.

We conducted a meta-analysis of sex-stratified GWASs. The meta-analysis was performed by the inverse variance-weighted method under the assumption of the fixed-effect model. The heterogeneity of effect sizes between sexes was evaluated by Cochran’s Q-test. We considered variants with *P* < 5.00 × 10^−8^ as significant in the GWAS.

We evaluated the association between the age at death and genotypes by linear regression analysis using sex, top 10 principal components, and affected diseases at baseline as covariates. We estimated *APOE* haplotypes by using hard call genotypes based on the imputed allelic dosages (*x*_dose_) of rs429358 and rs7412 (genotype = 0 for *x*_dos_ < 0.5; genotype = 2 for *x*_dose_ > 1.5; and genotype = 1 for 0.5 ≤ *x*_dose_ ≤ 1.5). We excluded nine individuals because their combination of genotypes (rs429358: CC and rs7412: CT) was not generally observed, and allelic dosages were halfway numbers (the ranges of allelic dosages were rs429358: 0.337–0.490 for T allele, and rs7412: 1.225–1.391 for C allele, respectively). Then, we subdivided them into ε2 (ε2ε3 and ε2ε3), ε3 (ε3ε3), and ε4 (ε4ε4 and ε4ε4), and performed an association analysis. R (ver.3.1.3) was used for the general analysis and illustration of figures.

### Pathway analysis

To investigate biological pathways associated with genetic factors related to survival time, we performed a gene-set analysis using Pascal^28^. Because this software uses linkage disequilibrium (LD) information, we generated tped-format genotype files from the reference panel used for the genotype imputation with PLINK (v1.9)^34^. We used gene sets reconstituted by DEPICT^27^ (Z-score threshold > 3) as proposed previously^35^. We calculated the FDR for the *P*-value for each gene set by the Benjamini–Hochberg method and regarded a gene set as significant if the FDR was <5%.

We evaluated the enrichment of significantly associated gene-set in patients with specific diseases. We selected five cancers (*N* > 3,000) and performed sex-stratified GWASs in these participants using SPACox software^27^. Then, gene-set enrichment analysis was performed in the same way.

### Functional annotation and pleiotropy

We used HaploReg^16^ (v4.1) to annotate functional information of the identified variants. We searched for eQTLs that were overlapped with genetic associations of newly identified locus using the publicly available dataset from GTEx^24^ (v6; a threshold for significant eQTL was a false discover rate less than 5%). The GTEx portal was used to identify variants suggested to have a protein-truncating effect^23,25^. We assessed the significance of overlap by considering whether the top hits of eQTLs were in LD (*r*^*2*^ > 0.7) with the variant identified by GWAS.

### Reporting summary

Further information on research design is available in the Nature Portfolio Reporting Summary linked to this article.

## Supplementary information

Supplementary Information

Description of Additional Supplementary Files

Supplementary Data 1

Supplementary Data 2

Supplementary Data 3

Supplementary Data 4

Reporting Summary

## Data Availability

The GWAS summary statistics are available in the National Bioscience Database Center (NBDC) Human Database (https://humandbs.biosciencedbc.jp/en/; dataset ID: hum0014; accession code: hum0014.v27.surv.v1) and jenger (http://jenger.riken.jp/en/) without restriction. The genotypes used in this study are available with accession codes JGAS00000000114 for the study and JGAD00000000123 for the genotypes from the Japanese Genotype-phenotype Archive (JGA; https://www.ddbj.nig.ac.jp/jga/index-e.html).

## Code availability

We used the following software to carry out our analysis: PLINK (https://www.cog-genomics.org/plink2); MACH (http://csg.sph.umich.edu//abecasis/MaCH/); SHAPEIT (https://mathgen.stats.ox.ac.uk/genetics_software/shapeit/shapeit.html); SPACox (https://github.com/WenjianBI/SPACox); Survival (https://cran.r-project.org/web/packages/survival/index.html); R (https://www.r-project.org/); LocusZoom (https://github.com/Geeketics/LocusZooms).
