# Supplementary material for: Genome-wide association study reveals BET1L associated with survival time in the 137,693 Japanese individuals
Source: Commun Biol. 2023 Feb 3;6:143. doi: 10.1038/s42003-023-04491-0 (PMC9898503; doi:10.1038/s42003-023-04491-0)
Supplement: Supplementary file 1 — Supplementary Information [file 42003_2023_4491_MOESM1_ESM.pdf]

Genome-wide association study reveals *BET1L* associated with survival time in the 137,693 Japanese individuals

Akiyama M et al.

Supplementary information:

Supplementary Figures (p.2–11)

Supplementary Fig.1 | QQ plot of sex-stratified GWAS.
Supplementary Fig.2 | Manhattan plot of sex-stratified GWAS.
Supplementary Fig.3 | Dominance effect of rs2280543.
Supplementary Fig.4 | Age at death stratified by rs2280543 genotype.
Supplementary Fig.5 | Pleiotropic effects of *BET1L* region across 219 traits.
Supplementary Fig.6 | Association plot of *BET1L* eQTL in muscle skeletal.
Supplementary Fig.7 | Effect sizes of rs76612380 stratified by affected diseases.
Supplementary Fig.8 | Causes of death stratified by rs2280543 genotype.
Supplementary Fig.9 | Survival curve after excluding individuals who died by cerebrovascular and cardiovascular death.
Supplementary Fig.10| Survival curve after stratification by *APOE* allele.

Supplementary Tables (p.12–18)

Supplementary Table 1 | Affected diseases at baseline.
Supplementary Table 2 | Characteristics of study subjects.
Supplementary Table 3 | Causes of death during follow-up.
Supplementary Table 4 | Epigenetic annotation of rs76612380.
Supplementary Table 5 | Pleiotropy of *BET1L.*
Supplementary Table 6 | Associations of *APOE* haplotypes.
Supplementary Table 7 | Enrichment of BCAR1 PPI subnetwork in patients with cancer.

Supplementary References (p.19)

a.

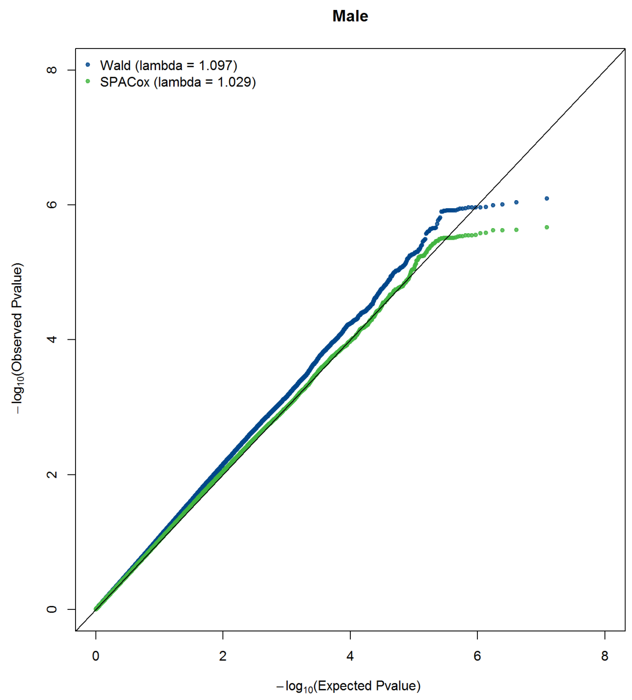

b.

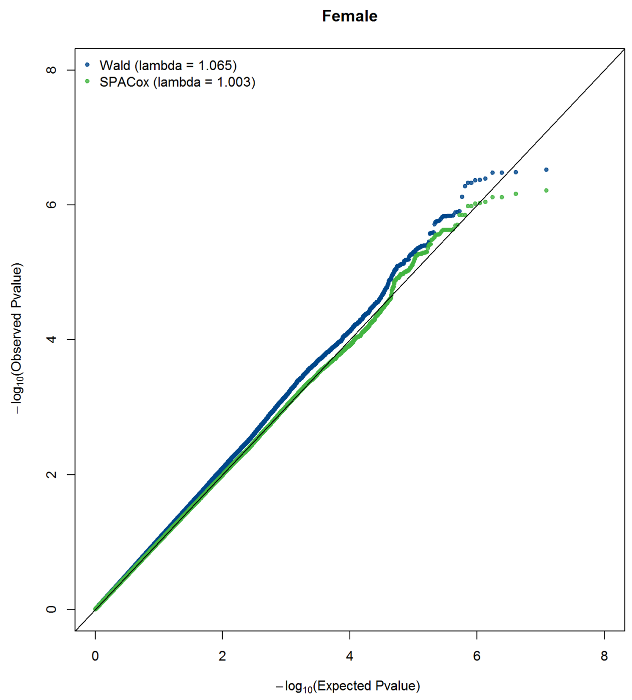

Supplementary Fig.1 | QQ plot of sex-stratified GWAS

QQ plots of GWAS for male (a) and female (b) are shown. We calculated P-values using Wald test (blue) and SPACox software (green), respectively.

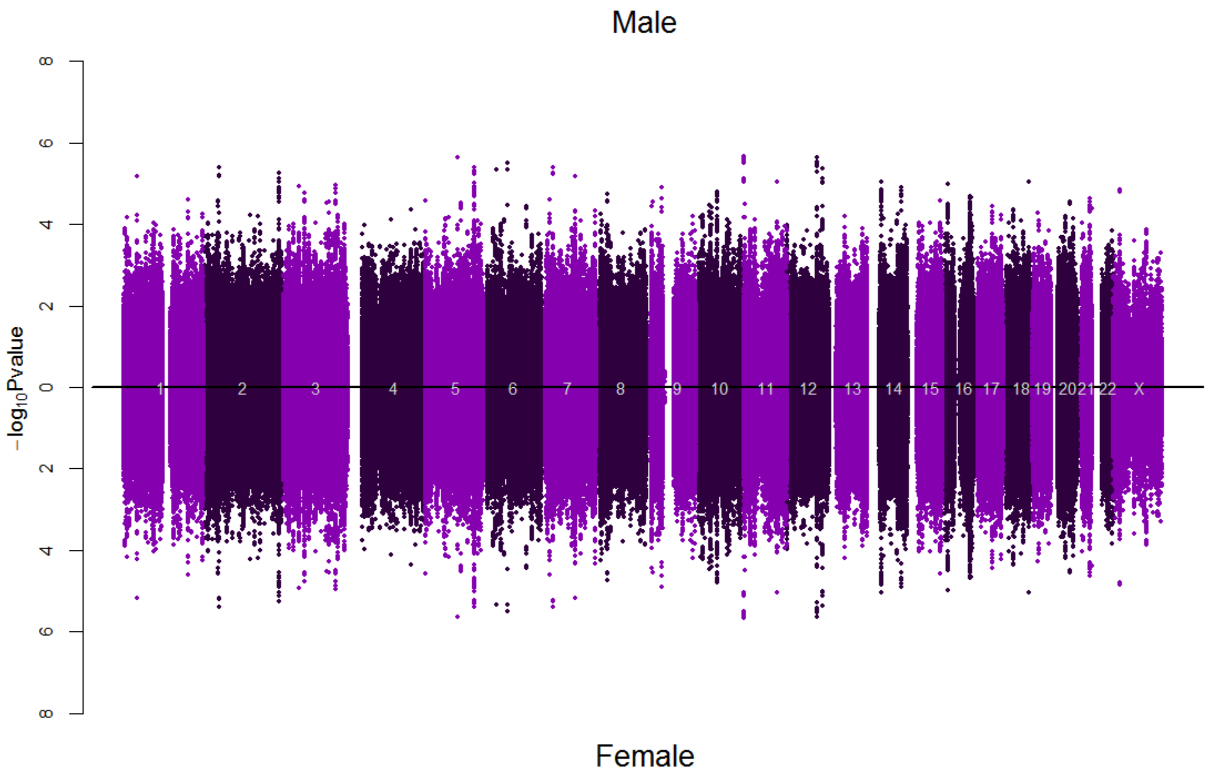

Supplementary Fig.2 | Manhattan plot of sex-stratified GWAS
The associations of sex-stratified GWASs are plotted. The associations of GWAS in male are plotted upper y-axis, and those in female GWAS are plotted lower y-axis.

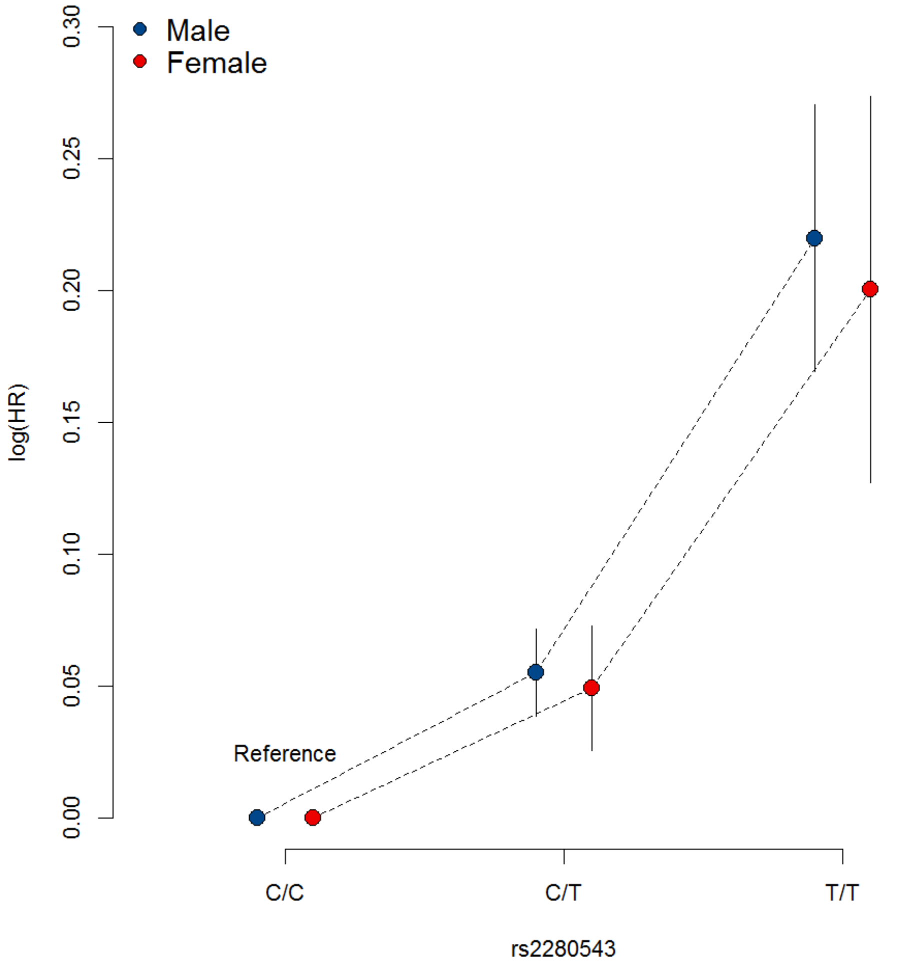

Supplementary Fig.3 | Dominance effect of rs2280543

We estimated the effect of C/T and T/T genotypes of rs2280543 on survival time using C/C genotype as reference in each sex. Y-axis denotes the effect size in log(HR) scale. Error bars indicate the standard errors of estimates. HR, hazard ratio.

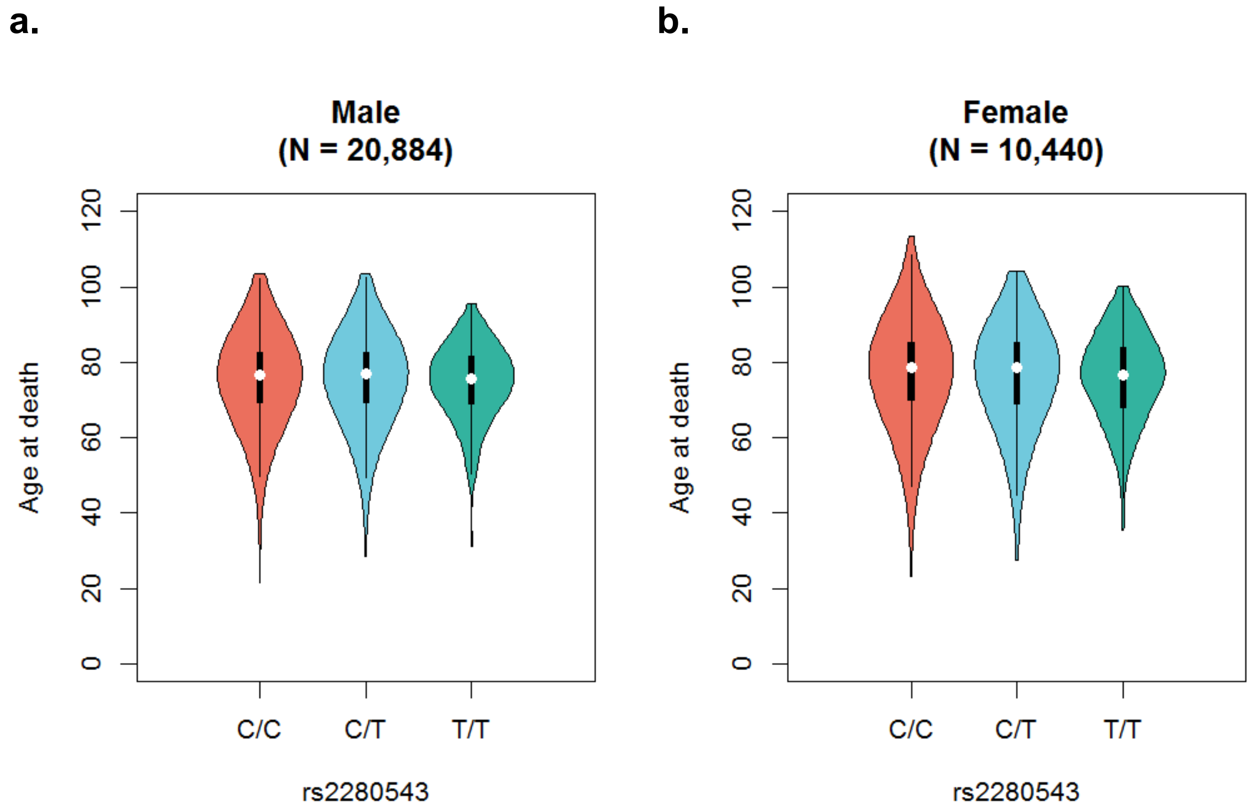

Supplementary Fig.4 | Age at death stratified by rs2280543 genotype
We compared the age at death stratified by rs2280543 genotypes in each sex. The violin plots denote the kernel densities and the box plots indicate the medians (shown as white dots), interquartile ranges (IQRs; shown as boxes) and 1.5 × IQRs (shown as lines) of age at death in the individuals who died during the follow-up period (*N* = 20,848 and 10,440 in male [a] and female [b], respectively).

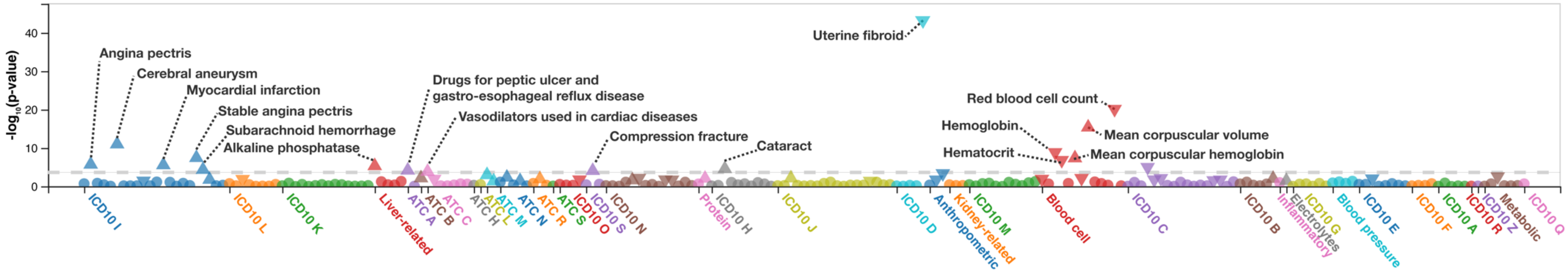

Supplementary Fig.5 | Pleiotropic effects of *BET1L* region across 219 traits

We plotted the associations of rs2280543 in the analyses of the Biobank Japan project (**Supplementary Ref.1**) using BioBank Japan PheWeb (https://pheweb.jp/).

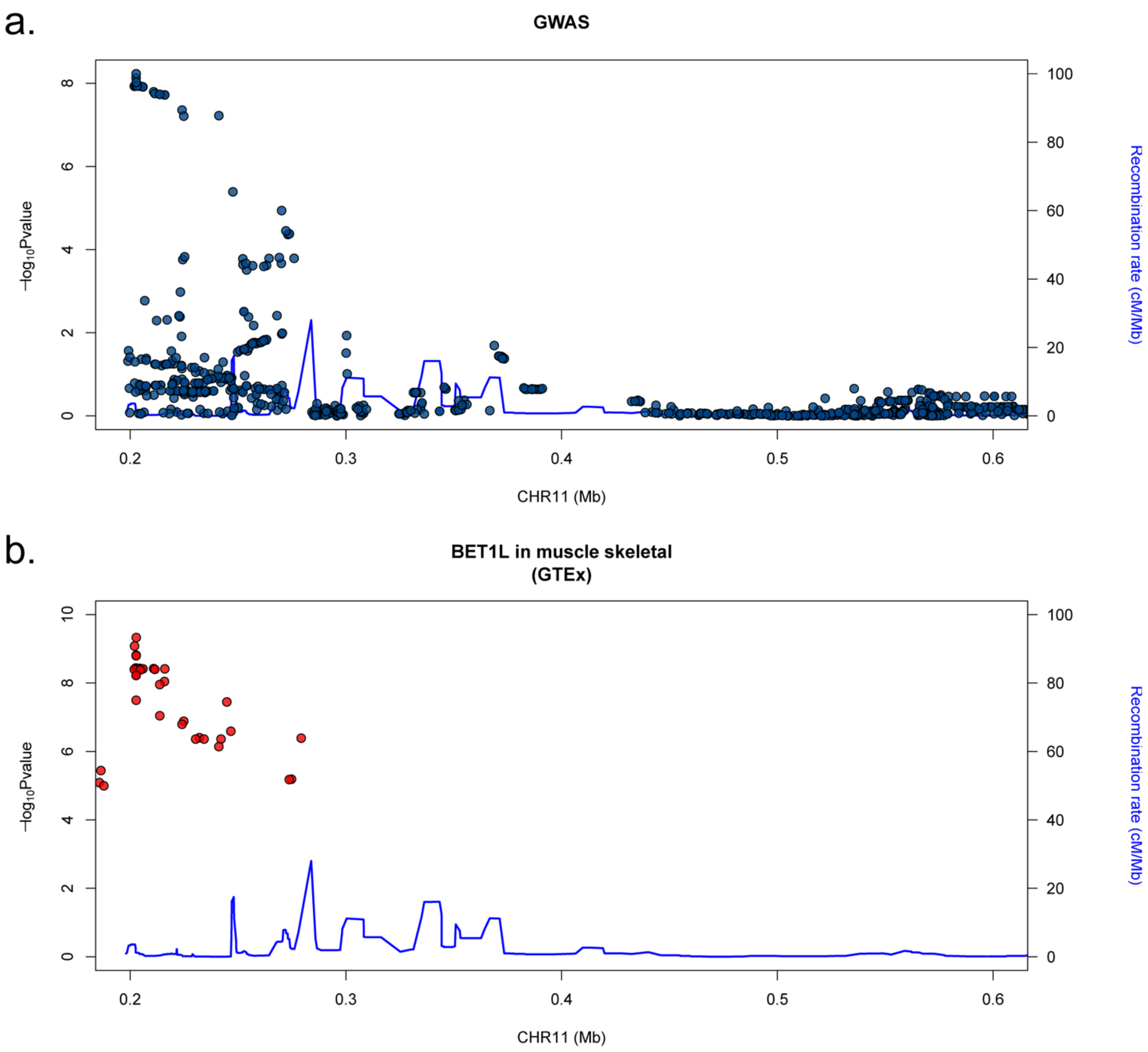

Supplementary Fig.6 | Association plot of *BET1L* eQTL in muscle skeletal
The upper panel shows the associations of meta-GWAS for survival in the Biobank Japan (a). We plotted the eQTL result for *BET1L* in GTEx project in muscle skeletal in lower panel (b). Similar association signals were found.

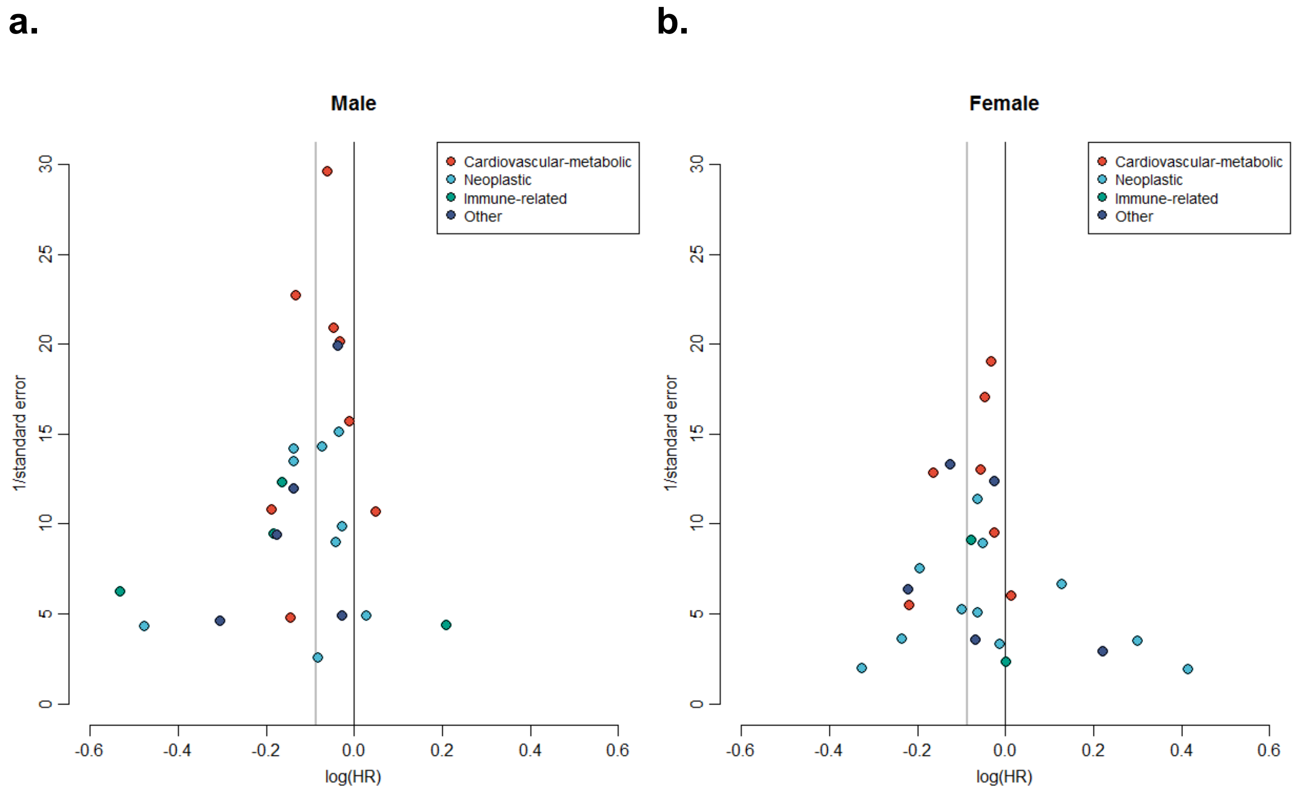

Supplementary Fig.7 | Effect sizes of rs76612380 stratified by affected diseases

We evaluated the effect size of rs76612380 by stratifying participants according to the disease status (N = 28 and 29 for male [a] and female [b], respectively; detailed in **Supplementary Table 1**). Each plot show the effect size in each stratified disease in log(HR) scale (x-axis; also shown in **Supplementary Data 1**) and its weight calculated by the inverse standard error (y-axis). The plots are colored according to the four disease groups. Gray vertical lines denote effect sizes estimated in each sex-stratified GWAS.

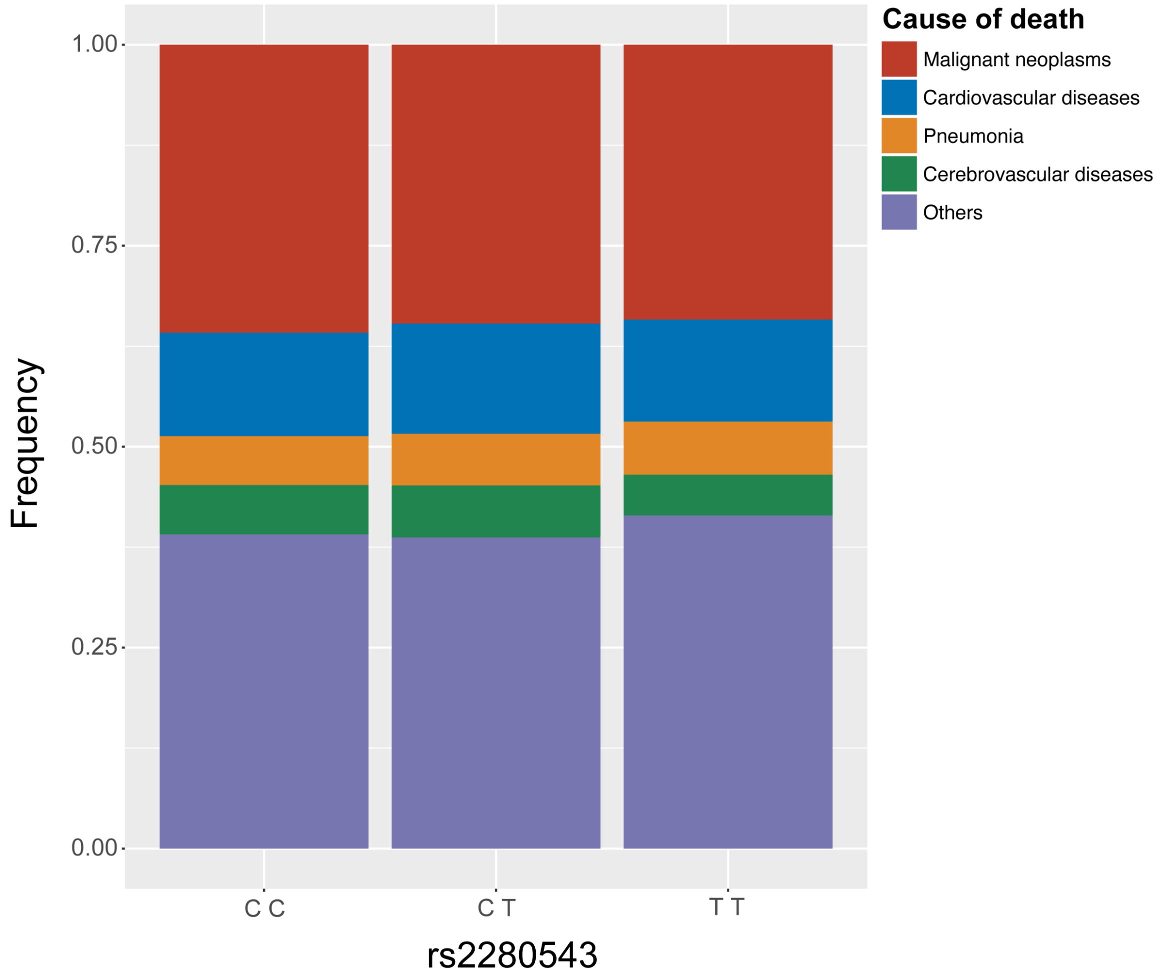

Supplementary Fig.8 | Causes of death stratified by rs2280543 genotype
We investigate the cases of death by dividing the participants based on rs2280543 genotypes.

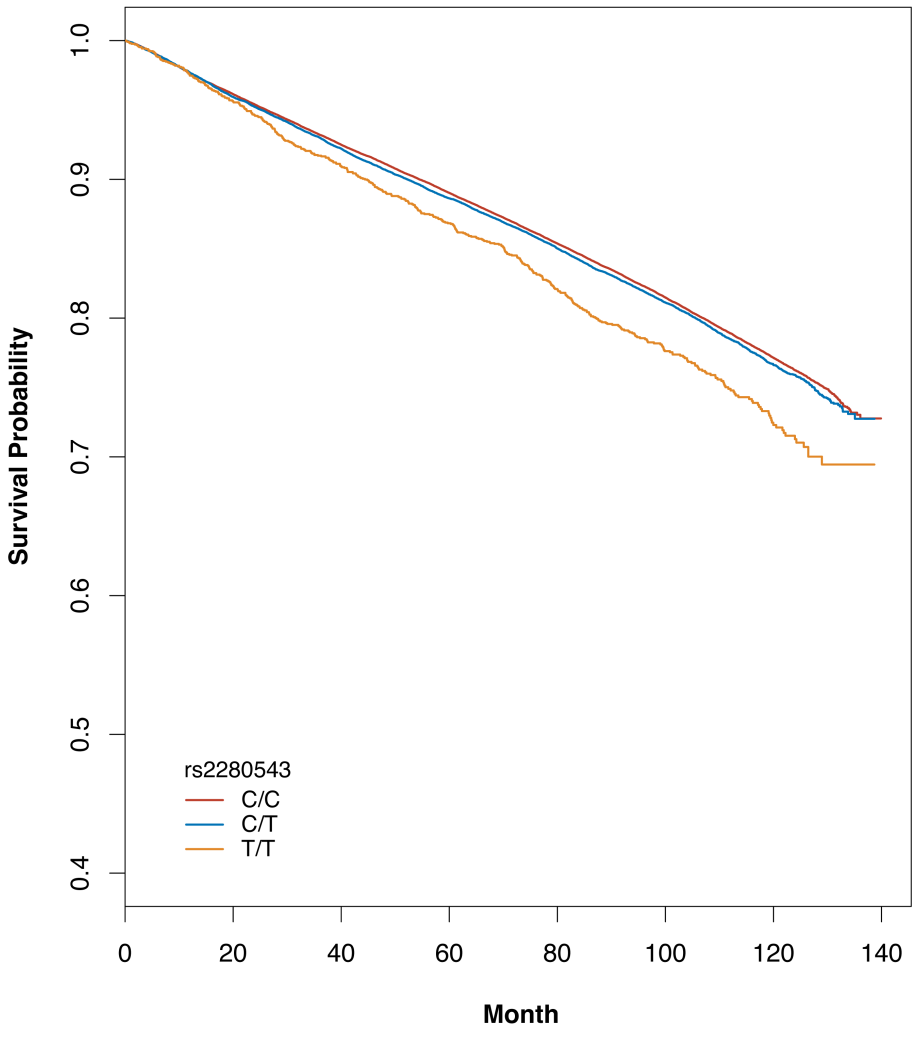

Supplementary Fig.9 | Survival curve after excluding individuals who died by cerebrovascular and cardiovascular death.

We evaluated the genetic effect of rs2280543 after excluding the individuals who died by cerebrovascular and cardiovascular diseases, and plotted the survival curve.

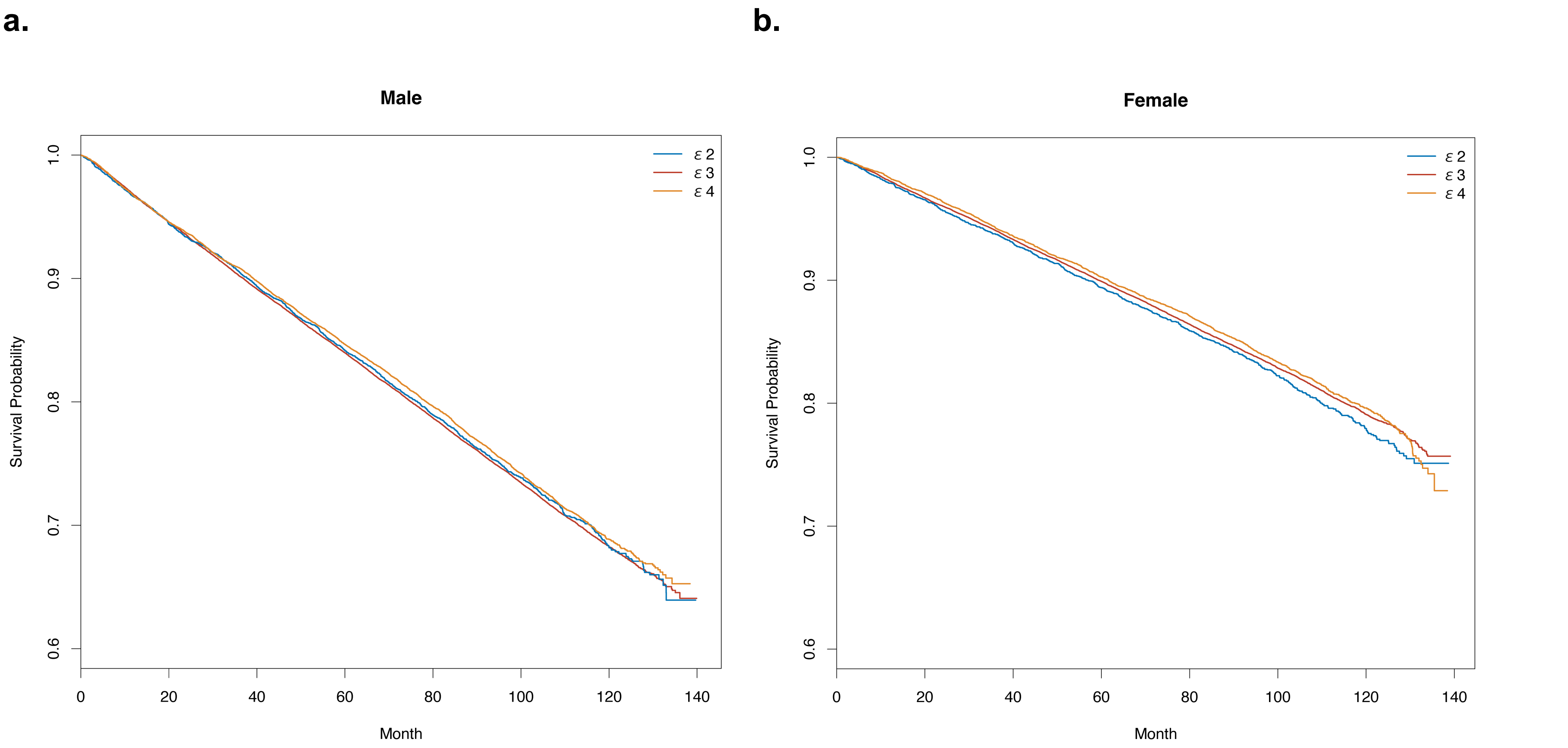

Supplementary Fig.10 | Survival curve after stratification by *APOE* allele

We evaluated the genetic effect of APOE allele after stratified participants into three groups (ε2 [ε2ε3 and ε2ε3], ε3 [ε3ε3], and ε4 [ε4ε4 and ε4ε4]) in each sex (male [a] and female [b]).

Supplementary Table 1 | Affected diseases at baseline

Disease	Disease category	Male (N= 78,029)		Female (N=59664)	
		N	%	N	%
Cerebral aneurysm	Cardiovascular-metabolic	815	1.04	1,513	2.54
Congestive heart failure	Cardiovascular-metabolic	4,156	5.33	2,455	4.11
Ischemic stroke	Cardiovascular-metabolic	8,960	11.48	5,203	8.72
Myocardial infarction	Cardiovascular-metabolic	9,674	12.40	2,185	3.66
Peripheral artery disease	Cardiovascular-metabolic	1,846	2.37	506	0.85
Stable angina pectoris	Cardiovascular-metabolic	9,172	11.75	3,820	6.40
Unstable angina pectoris	Cardiovascular-metabolic	2,809	3.60	942	1.58
Type 2 diabetes	Cardiovascular-metabolic	22,095	28.32	12,417	20.81
Dyslipidemia	Cardiovascular-metabolic	19,826	25.41	18,681	31.31
Atopic dermatitis	Immune-related	123	0.16	125	0.21
Asthma	Immune-related	3,222	4.13	3,498	5.86
Drug eruption	Immune-related	141	0.18	126	0.21
Pollinosis	Immune-related	477	0.61	601	1.01
Graves' disease	Immune-related	189	0.24	422	0.71
Rheumatoid arthritis	Immune-related	740	0.95	2,996	5.02
Chronic hepatitis B	Immune-related	753	0.97	422	0.71
Chronic hepatitis C	Immune-related	2,750	3.52	2,317	3.88
Pulmonary tuberculosis	Immune-related	254	0.33	95	0.16
Breast cancer	Neoplastic	23	0.03	5,010	8.40
Biliary tract cancer	Neoplastic	124	0.16	89	0.15
Cervical cancer	Neoplastic	0	0.00	540	0.91
Colorectal cancer	Neoplastic	3,737	4.79	2,174	3.64
Endometrial cancer	Neoplastic	0	0.00	888	1.49
Esophageal cancer	Neoplastic	952	1.22	132	0.22
Gastric cancer	Neoplastic	4,028	5.16	1,409	2.36
Hematological malignancy	Neoplastic	557	0.71	450	0.75
hepatocellular carcinoma	Neoplastic	994	1.27	305	0.51
Lung cancer	Neoplastic	2,156	2.76	1,171	1.96
Ovarian cancer	Neoplastic	0	0.00	646	1.08
Pancreatic cancer	Neoplastic	178	0.23	92	0.15
Prostate cancer	Neoplastic	4,546	5.83	0	0.00
Cataract	Other	3,880	4.97	4,151	6.96
Cirrhosis	Other	870	1.11	509	0.85

Chronic obstructive pulmonary disease	Other	1,949	2.50	288	0.48
Endometriosis	Other	0	0.00	146	0.24
Epilepsy	Other	394	0.50	219	0.37
Glaucoma	Other	822	1.05	897	1.50
Interstitial lung disease	Other	261	0.33	185	0.31
Keloid	Other	63	0.08	66	0.11
Nephrotic syndrome	Other	251	0.32	172	0.29
Osteoporosis	Other	410	0.53	5,176	8.68
Periodontal disease	Other	145	0.19	119	0.20
Uterine fibroids	Other	0	0.00	837	1.40
Urolithiasis	Other	909	1.16	308	0.52
Arrhythmia	Other	9,129	11.70	4,973	8.34

The baseline information on affected diseases are shown.

Supplementary Table 2 | Characteristics of study subjects

	Male	Female
N	78,029	59,664
Age	64.7 ± 11.8	64.9 ± 12.7
Follow-up period (month)	87.1 ± 35.5	92.1 ± 32.6

The baseline characteristics of participants are summarized.

Supplementary Table 3 | Causes of death during follow-up

	Male	Female
Number of death (%)	20,884 (26.8%)	10,440 (17.5%)
Cause of death		
- Malignant neoplasm	7,571 (36.3%)	3,558 (34.1%)
- Cardiovascular disease	2,692 (12.9%)	1,396 (13.4%)
- Pneumonia	1,433 (6.9%)	504 (4.8%)
- Cerebrovascular disease	1,262 (6.0%)	675 (6.5%)
- Others	7,926 (38.0%)	4,307 (41.3%)

All causes of death were determined as we described previously (**Supplementary Ref.2**).

Supplementary Table 4 | Epigenetic annotation of rs76612380

Promoter histone marks (2_TssAFlnk)	Enhancer histone marks (6_EnhG and 7_Enh)
Primary hematopoietic stem cells short term culture	H1 Derived Mesenchymal Stem Cells
Primary T helper cells PMA-I stimulated	Primary B cells from cord blood
Primary hematopoietic stem cells G-CSF-mobilized Female	Primary T cells from peripheral blood
Primary hematopoietic stem cells G-CSF-mobilized Male	Primary T helper 17 cells PMA-I stimulated
Monocytes-CD14+ RO01746 Primary Cells	Primary T regulatory cells from peripheral blood
	Primary mononuclear cells from peripheral blood
	Adipose Nuclei
	Duodenum Mucosa
	Fetal Adrenal Gland
	Placenta
	Spleen
	K562 Leukemia Cells
	hESC Derived CD184+ Endoderm Cultured Cells
	Primary monocytes from peripheral blood
	Primary neutrophils from peripheral blood
	Primary T cells from cord blood
	Primary hematopoietic stem cells
	Primary T helper memory cells from peripheral blood 2
	Primary T helper naive cells from peripheral blood
	Primary T helper naive cells from peripheral blood
	Primary T helper memory cells from peripheral blood 1
	Primary T helper cells from peripheral blood
	Primary T cells effector/memory enriched from peripheral blood
	Primary T CD8+ naive cells from peripheral blood
	Primary T CD8+ memory cells from peripheral blood
	Foreskin Melanocyte Primary Cells skin01
	Fetal Thymus
	Thymus

We used HaploReg (v4.1) for the epigenetic annotation. The cell types which contain promoter or enhancer marks (estimatd by ChrommHMM 15 states model) overlapped with rs76612380 are shown.

Supplementary Table 5 | Pleiotropy of *BET1L*

Category	Phenotype	Number of samples	P-value	Effect Size (se)	Lead variant	POS	r2 with rs2280543
ICD10 D	Uterine fibroid	14,475 / 65,733	5.9E-44	-0.30 (0.021)	rs12225799	241124	0.996
Blood cell	Red blood cell count	153,512	7.5E-21	-0.044 (0.0047)	rs11245990	202565	1.000
Blood cell	Mean corpuscular volume	129,832	4.1E-16	0.044 (0.0054)	rs12226697	216056	0.987
ICD10 I	Cerebral aneurysm	3,132 / 152,022	1.0E-11	0.27 (0.040)	rs73392700	224845	0.996
Blood cell	Hemoglobin	152,447	1.4E-09	-0.028 (0.0046)	11:201922:T:C	201922	0.996
ICD10 I	Stable angina pectoris	18,833 / 146,214	3.6E-08	0.098 (0.018)	rs12225799	241124	0.996
Blood cell	Mean corpuscular hemoglobin	128,028	4.2E-08	0.030 (0.0055)	rs12226697	216056	0.987
Blood cell	Hematocrit	153,015	1.5E-07	-0.025 (0.0047)	rs12226698	213740	1.000
ICD10 I	Angina pectoris	14,007 / 145,158	1.9E-06	0.095 (0.020)	rs12225799	241124	0.996
ICD10 I	Myocardial infarction	14,992 / 146,214	2.7E-06	0.094 (0.020)	rs117629395	229439	0.684
Liver-related	Alkaline phosphatase	118,886	4.7E-06	0.027 (0.0058)	rs3817640	297970	0.003
ICD10 C	Colorectal cancer	8,305 / 159,386	9.8E-06	-0.11 (0.025)	11:244817:GA:G	244817	0.996
ICD10 H	Cataract	38,194 / 140,532	2.7E-05	0.057 (0.014)	11:244817:GA:G	244817	0.996
ICD10 I	Subarachnoid hemorrhage	1,203 / 152,022	4.5E-05	0.26 (0.063)	rs554773	247630	0.731
ATC A	Drugs for peptic ulcer and gastro-esophageal reflux disease	53,363 / 125,363	6.7E-05	0.044 (0.011)	rs3782121	205935	1.000
ICD10 S	Compression fracture	2,856 / 175,870	8.5E-05	0.16 (0.041)	rs11246271	701503	0.000
ATC C	Vasodilators used in cardiac diseases	17,050 / 161,676	1.4E-04	0.069 (0.018)	rs11246002	211447	0.975

To assess pleiotropic associations of identified locus in the Japanese population, we looked up the statistics of rs2280543 in the catalog of genetic associations across 219 traits (**Supplementary Ref.1**). Seventeen traits which showed significant associations at Bonferroni-level are shown in the table.

Supplementary Table 6 | Associations of *APOE* haplotypes.

APOE allele	Male			Female			Meta-analysis		
	N	HR (95%CI)	*P*-value	N	HR (95%CI)	*P*-value	HR (95%CI)	*P*-value	P_{het}
ε2	6,097	0.92 (0.87-1.09)	9.01×10^{-4}	4,551	0.99 (0.92-1.06)	0.70	0.94 (0.90-0.98)	3.42×10^{-3}	0.11
ε3	57,723	Reference		44,446	Reference		Reference		
ε4	14,203	1.03 (0.99-1.06)	0.16	10,664	1.11 (1.06-1.17)	5.46×10^{-5}	1.05 (1.02-1.08)	5.11×10^{-4}	0.01

Based on the genotypes obtained from imputed allelic dosage, we estimated *APOE* haplotypes. We subdivided them into ε2 (ε2ε3 and ε2ε3), ε3 (ε3ε3), and ε4 (ε4ε4 and ε4ε4), and performed an association analysis.

HR, hazard ratio; CI, confidence interval；P_{het}, *P*-value for heterogeneity.

Supplementary Table 7 | Enrichment of *BCAR1* PPI subnetwork in patients with cancer.

Disease	Sex	*P*-value
Lung cancer	Male	0.66
	Female	0.08
Breast cancer	Female	0.98
Gastric cancer	Male	0.10
	Female	0.42
Colorectal cancer	Male	0.60
	Female	0.90
Prostate cancer	Male	0.44

We estimated P-value for enrichment of BCAR1 protein-protein subnetwork using PASCAL after performing GWAS in participants with cancer. We selected five diseases with sample sizes > 3,000. Number of samples are shown in Supplementary Table 1.

Supplementary References:

1. Sakaue, S. *et al.* A cross-population atlas of genetic associations for 220 human phenotypes. *Nat. Genet.* **53,** 1415–1424 (2021).

2. Hirata, M. *et al.* Overview of BioBank Japan follow-up data in 32 diseases. *J. Epidemiol.* **27,** 22–28 (2017).
