# Supplementary material for: Genome-wide association study reveals BET1L associated with survival time in the 137,693 Japanese individuals
Source: Commun Biol. 2023 Feb 3;6:143. doi: 10.1038/s42003-023-04491-0 (PMC9898503; doi:10.1038/s42003-023-04491-0)
Supplement: Supplementary file 2 — Description of Additional Supplementary Files [file 42003_2023_4491_MOESM2_ESM.pdf]

Description of Additional Supplementary Files

File name: Supplementary Data 1

Description: Result of sensitivity analysis

File name: Supplementary Data 2

Description: Associations at previously implicated variants

File name: Supplementary Data 3

Description: Result of gene-set enrichment analysis

File name: Supplementary Data 4

Description: Gene level association of BCAR1 PPI subnetwork
