# Supplementary material for: Genome-wide association study reveals BET1L associated with survival time in the 137,693 Japanese individuals
Source: Commun Biol. 2023 Feb 3;6:143. doi: 10.1038/s42003-023-04491-0 (PMC9898503; doi:10.1038/s42003-023-04491-0)
Supplement: Supplementary file 7 — Reporting Summary [file 42003_2023_4491_MOESM7_ESM.pdf]

nature research

Corresponding author(s): Yoichiro Kamatani

Last updated by author(s): Oct 3, 2022

Reporting Summary

Nature Research wishes to improve the reproducibility of the work that we publish. This form provides structure for consistency and transparency in reporting. For further information on Nature Research policies, see our Editorial Policies and the Editorial Policy Checklist.

Statistics

For all statistical analyses, confirm that the following items are present in the figure legend, table legend, main text, or Methods section.

n/a	Confirmed	
☐	☒	The exact sample size (n) for each experimental group/condition, given as a discrete number and unit of measurement
☒	☐	A statement on whether measurements were taken from distinct samples or whether the same sample was measured repeatedly
☐	☒	The statistical test(s) used AND whether they are one- or two-sided<br>*Only common tests should be described solely by name; describe more complex techniques in the Methods section.*
☐	☒	A description of all covariates tested
☐	☒	A description of any assumptions or corrections, such as tests of normality and adjustment for multiple comparisons
☐	☒	A full description of the statistical parameters including central tendency (e.g. means) or other basic estimates (e.g. regression coefficient) AND variation (e.g. standard deviation) or associated estimates of uncertainty (e.g. confidence intervals)
☐	☒	For null hypothesis testing, the test statistic (e.g. F, t, r) with confidence intervals, effect sizes, degrees of freedom and P value noted<br>*Give P values as exact values whenever suitable.*
☒	☐	For Bayesian analysis, information on the choice of priors and Markov chain Monte Carlo settings
☒	☐	For hierarchical and complex designs, identification of the appropriate level for tests and full reporting of outcomes
☐	☒	Estimates of effect sizes (e.g. Cohen's d, Pearson's r), indicating how they were calculated

Our web collection on statistics for biologists contains articles on many of the points above.

Software and code

Policy information about availability of computer code

Data collection	We do not use software for data collection.
Data analysis	We used principal component analyses of genotype data using in-house software (ref.29,30). We phased genotypes using MACH and imputed the variants using Minimac (v0.1.1). 'Survival' package in R was used for survival analysis. Pascal was used for gene-set enrichment analysis. We used HaploReg (v4.1) to annotate functional information of the identified variants. PLINK (v1.9) and R (ver.3.1.3) were used for the general analysis and illustration of figures.

For manuscripts utilizing custom algorithms or software that are central to the research but not yet described in published literature, software must be made available to editors and reviewers. We strongly encourage code deposition in a community repository (e.g. GitHub). See the Nature Research guidelines for submitting code & software for further information.

Data

Policy information about availability of data

All manuscripts must include a data availability statement. This statement should provide the following information, where applicable:

- Accession codes, unique identifiers, or web links for publicly available datasets
- A list of figures that have associated raw data
- A description of any restrictions on data availability

The GWAS summary statistics will be deposited in the National Bioscience Database Center (https://humandbs.biosciencedbc.jp/en/) and jenger (http://jenger.riken.jp/) and will be available without restriction. The genotype data employed in the GWAS are available from the Japanese Genotype-phenotype Archive (JGA; http://trace.ddbj.nig.ac.jp/jga/index_e.html) with accession codes JGAS00000000114 for the study.

Field-specific reporting

Please select the one below that is the best fit for your research. If you are not sure, read the appropriate sections before making your selection.

☒ Life sciences ☐ Behavioural & social sciences ☐ Ecological, evolutionary & environmental sciences

For a reference copy of the document with all sections, see nature.com/documents/nr-reporting-summary-flat.pdf

Life sciences study design

All studies must disclose on these points even when the disclosure is negative.

Sample size	We included 141,612 participants of the Biobank Japan project with 32 systemic diseases as much as possible.
Data exclusions	We excluded individuals < 20 years old and participants with amyotrophic lateral sclerosis.
Replication	We do not have replication samples.
Randomization	Not applicable
Blinding	Not applicable

Reporting for specific materials, systems and methods

We require information from authors about some types of materials, experimental systems and methods used in many studies. Here, indicate whether each material, system or method listed is relevant to your study. If you are not sure if a list item applies to your research, read the appropriate section before selecting a response.

Materials & experimental systems

n/a	Involved in the study	
☒	☐	Antibodies
☒	☐	Eukaryotic cell lines
☒	☐	Palaeontology and archaeology
☒	☐	Animals and other organisms
☐	☒	Human research participants
☐	☒	Clinical data
☒	☐	Dual use research of concern

Methods

n/a	Involved in the study	
☒	☐	ChIP-seq
☒	☐	Flow cytometry
☒	☐	MRI-based neuroimaging

Human research participants

Policy information about studies involving human research participants

Population characteristics	We summarized the baseline characteristics of the participants in Supplementary Tables 1 and 2.
Recruitment	We used the follow-up data of the BBJ project.
Ethics oversight	This study was approved by the Ethics Committees of the participating hospitals, the University of Tokyo, and RIKEN.

Note that full information on the approval of the study protocol must also be provided in the manuscript.

Clinical data

Policy information about clinical studies

All manuscripts should comply with the ICMJE guidelines for publication of clinical research and a completed CONSORT checklist must be included with all submissions.

Clinical trial registration	Not applicable.
Study protocol	Not applicable.
Data collection	We used clinical data obtained from the follow-up survey of the Biobank Japan project from 2010 to 2014.
Outcomes	All cause mortality of the participants.
